# Noninvasive Assessment of Hepatitis C Virus Infected Patients Using Vibration-Controlled Transient Elastography

**DOI:** 10.3390/jcm10122575

**Published:** 2021-06-10

**Authors:** Mira Florea, Teodora Serban, George Razvan Tirpe, Alexandru Tirpe, Monica Lupsor-Platon

**Affiliations:** 1 Community Medicine Department, Iuliu Hatieganu University of Medicine and Pharmacy, 400012 Cluj-Napoca, Romania; miraflorea@umfcluj.ro; 2 Medical Imaging Department, Iuliu Hatieganu University of Medicine and Pharmacy, 400012 Cluj-Napoca, Romania; serban.teodora8@gmail.com; 3 Department of Radiology and Medical Imaging, County Emergency Hospital Cluj-Napoca, 3-5 Clinicilor Street, 400000 Cluj-Napoca, Romania; razvantirpe@gmail.com; 4 Research Center for Functional Genomics, Biomedicine and Translational Medicine, Iuliu Hatieganu University of Medicine and Pharmacy, 23 Marinescu Street, 400337 Cluj-Napoca, Romania; altirpe@gmail.com; 5 Medical Imaging Department, Regional Institute of Gastroenterology and Hepatology, 400162 Cluj-Napoca, Romania

**Keywords:** chronic hepatitis C, vibration controlled transient elastography, fibrosis, steatosis, hepatocellular carcinoma

## Abstract

Chronic infection with hepatitis C virus (HCV) is one of the leading causes of cirrhosis and hepatocellular carcinoma (HCC). Surveillance of these patients is an essential strategy in the prevention chain, including in the pre/post-antiviral treatment states. Ultrasound elastography techniques are emerging as key methods in the assessment of liver diseases, with a number of advantages such as their rapid, noninvasive, and cost-effective characters. The present paper critically reviews the performance of vibration-controlled transient elastography (VCTE) in the assessment of HCV patients. VCTE measures liver stiffness (LS) and the ultrasonic attenuation through the embedded controlled attenuation parameter (CAP), providing the clinician with a tool for assessing fibrosis, cirrhosis, and steatosis in a noninvasive manner. Moreover, standardized LS values enable proper staging of the underlying fibrosis, leading to an accurate identification of a subset of HCV patients that present a high risk for complications. In addition, VCTE is a valuable technique in evaluating liver fibrosis prior to HCV therapy. However, its applicability in monitoring fibrosis regression after HCV eradication is currently limited and further studies should focus on extending the boundaries of VCTE in this context. From a different perspective, VCTE may be effective in identifying clinically significant portal hypertension (CSPH). An emerging prospect of clinical significance that warrants further study is the identification of esophageal varices. Our opinion is that the advantages of VCTE currently outweigh those of other surveillance methods.

## 1. Introduction

The global estimates of hepatitis C virus (HCV) infection appraised chronic hepatitis C (CHC) as one of the leading causes of cirrhosis and hepatocellular carcinoma (HCC), with an approximate global prevalence of HCV infection at 1.6% [1,2]. Specifically, CHC patients may silently develop cirrhosis in up to 20% of cases. In addition, patients with CHC and cirrhosis may develop HCC in up to 5% of cases per year [3]. HCV transmission routes are dependent on blood and blood products [4]. The diagnosis of HCV infection can be achieved through serologic assays and molecular RNA-based assays. In general terms, third generation serologic assays have a sensitivity of over 99% when CHC is suspected [4]. However, the silent progression of CHC towards cirrhosis prompts for new diagnostic means that can identify this pathological tendency early on the evolution axis. Liver fibrosis (LF) staging is paramount as it carries multiple roles—it is essential for the antiviral therapy, in the management of individuals after successful HCV treatment, and for prognosis purposes [5]. In addition, steatosis can accelerate liver fibrosis progression in HCV patients, and is associated with lower virologic response to antiviral therapy [6]. Although there is evidence of the contribution of ultrasound and even of artificial intelligence-enhanced US image analysis in steatosis quantification [7], new imaging techniques such as elastography are considered an essential add-on. The highly efficient direct-acting antiviral (DAA) therapies and noninvasive measures of liver fibrosis are two scientific advances that changed the management of patients with chronic HCV infection in the last decade [8].

Liver biopsy (LB) is an invasive method for staging fibrosis and grading steatosis and necroinflammatory activity [1]. It presents a number of drawbacks, including the risk of serious complications that may influence the patient acceptance rate and the lack of dynamic evaluation of liver fibrosis in time [9,10]. Although LB remains the reference standard for assessing necroinflammation and fibrosis, its limitations as an invasive procedure and requires repeated sampling, which has led to the use and development of several other noninvasive test as alternatives [11].

Conventional ultrasonography (US) (with or without contrast enhancement) is a noninvasive, cost-effective, widely available, and rapid technique that enables the examination of patients with chronic liver diseases (CLD) [12]. By evaluating structural changes, US proved to be particularly useful for the detection of cirrhosis and focal liver lesions (FLL) [12,13]. However, US fails to discern between lower stages of fibrosis, in which has led to the introduction of US elastography in order to overcome this drawback [14].

Vibration-controlled transient elastography (VCTE) is a novel, noninvasive, cost-efficient method for fibrosis staging using liver stiffness measurement (LSM) [10]. Furthermore, through the embedded Controlled Attenuation Parameter (CAP) tool, VCTE is able to simultaneously assess liver steatosis by estimating the total ultrasonic attenuation [15]. The current tendency of liver fibrosis assessment leans in favor of VCTE, as ultrasound elastography methods are becoming the standard of care in comparison to liver biopsy [1].

The present review aims to explore the current status of VCTE as noninvasive imaging assessment tool of HCV-infected patients through the lens of evidence-based medicine, underlining the differences between VCTE and conventional US.

## 2. The Principle of Vibration-Controlled Transient Elastography (VCTE, TE)

As previously mentioned, VCTE is a quantitative method for the noninvasive assessment of liver stiffness. It is composed of a device with readout—FibroScan^®^ (Echosens, Paris, France)—and different types of probes (S, M and XL). Choosing the correct transducer, according to the circumference of the patients’ thorax, is an important step in order to have a successful examination. While a circumference lower than 75 cm indicates the use of the S probe, the M probe is indicated for a circumference of over 75 cm. Furthermore, if the distance between the skin and liver capsule is greater than 25 mm, the XL transducer is the preferred option. It is worth mentioning that the median liver stiffness is significantly lower with XL probe compared to the M probe [16].

The ideal VCTE examination takes place with a patient who has fasted for 3 h prior to the measurement [17,18]. Depending on the thickness of the abdominal wall, one of the handheld probes is chosen and, together with the applied conduction gel, the probe is placed intercostally overlying the right hepatic lobe [19,20,21]. The probe generates a vibration wave, which travels through the liver and simultaneously receives ultrasound waves, calculating liver stiffness, rendered in kilopascal (kPa). In order to provide a median value of LS, ten successful measurements are required. LS can range widely between 2.5–75 kPa, with normal values being around 5 kPa. LS does not absolutely stage fibrosis like a biopsy would, but high values are significantly correlated with histology and are able to provide a risk estimate for advanced liver disease [22].

Simultaneously, the CAP (measured in dB/m) is calculated based on the attenuation of the ultrasound signal, with the purpose of evaluating the underlying liver steatosis in a noninvasive manner [23]. Chon et al. [24] suggested that the range of normal CAP values within the 5th–95th percentiles was 156.0–287.8 dB/m, with gender, body mass index, diabetes, and etiology independently affecting CAP values [25].

## 3. Pathological Changes Influencing Liver Stiffness

A comprehensive evaluation of the factors that increase liver stiffness is considered paramount. In a study by Lupsor et al. [26] that included 324 HCV patients, the authors found a strong correlation between LS and different histopathological parameters such as fibrosis (r = 0.759, *p* < 0.0005), necroinflammatory activity (r = 0.378, *p* < 0.0005), and steatosis (r = 0.255, *p* < 0.0005). Among these three, however, the stage of fibrosis is the single most important predictor.

Nevertheless, ingestion of food prior to LS measurement is another reason for increased kPa values. In a study by Arena et al. [17], LS was evaluated following a standardized meal in 125 confirmed HCV patients at different stages of fibrotic evolution. An elevation in kPa values was observed 15 to 45 minutes after ingestion of the meal and was higher among patients with increased stages of fibrosis (*p* < 0.001) and maximal among those with cirrhosis. Other factors that influence liver stiffness irrespective of fibrosis are mechanic cholestasis, central venous pressure and congestion, portal or arterial pressure, alcohol consumption, water retention, Valsalva and orthostatic maneuvers, as well as amyloidosis [27,28].

A rise in LS values along with a rise in ALT levels can be detected in patients with hepatitis due to cellular swelling and cholestasis. Furthermore, the increased stiffness values identified in patients with relapsed chronic hepatitis are not only found due to fibrosis, but also due to the superimposed cellular intumescence [29]. In a study by Bota et al. [30], the LS cutoffs were significantly higher in patients with increased ALT levels between 1.1 and 5-fold the standard value compared to those with normal ALT levels, 12.3 kPa versus 9.1 kPa, respectively. Consequently, caution must be taken when assessing liver stiffness in patients with increased ALT values because there is a risk of overestimating the stage of fibrosis [16].

## 4. Fibrosis Assessment by VCTE in HCV-Infected Patients

Among patients with CHC, determining liver fibrosis stage is essential for prognosis, follow-up, and antiviral therapy [5]. The European Federation of Societies for Ultrasound in Medicine and Biology (EFSUMB) guidelines outline that the two clinically relevant endpoints in HCV patients are the detection of significant fibrosis and, above anything else, the detection of cirrhosis [27]

As previously implied, the widely available US method fails to discern fibrosis in its early stages, which led to the introduction of novel elastography technologies. In fact, a recent study by Zhang et al. [13] found VCTE to be superior to US for the detection of significant fibrosis (AUROC, 0.84 versus 0.73; *p* = 0.02), advanced fibrosis (AUROC, 0.95 versus 0.76; *p* < 0.001), and cirrhosis (AUROC, 0.96 versus 0.71; *p* < 0.001) in a cohort of 94 patients with chronic hepatitis B and nonalcoholic fatty liver disease. In addition, the combination of VCTE and US did not increase the diagnostic accuracy for neither of these stages, compared to VCTE alone. However, their association significantly improved the specificity (95.7% versus 76.6%, *p* < 0.001) and positive predictive value (94.3% versus 77.1%, *p* = 0.002) in contrast to VCTE alone. Similar results were observed by Wang et al. [31] in 320 patients with chronic viral hepatitis. Regarding other noninvasive methods, an evidence-based analysis concluded that neither FibroTest, nor acoustic radiation force impulse were superior to VCTE [32].

HCV infected patients are the first to have benefited from VCTE. Several studies reported excellent diagnostic accuracy of VCTE for the detection of fibrosis in HCV patients. As exemplified in Table 1, LS significantly correlates with the degree of liver fibrosis assessed by LB, even if some adjacent stages tend to overlap [10,26,33,34,35,36,37,38,39,40,41,42,43]. The AUROC values range from 0.838 to 0.936 for incipient fibrosis (≥F1), 0.690 to 0.91 for significant fibrosis (≥F2), 0.737 to 0.99 for advanced fibrosis (≥F3), and 0.852 to 0.99 for cirrhosis (F4) prediction, at cutoff values of 5.3–5.5 kPa (≥F1), 4.5–8.8 kPa (≥F2), 9.1–11 kPa (≥F3) and 11.3–16.9 kPa (F4), respectively. These values range significantly, mainly because of the varying prevalence of fibrosis stage in each study group along with the particular diagnostic aims of the investigation [44]. Thereby, the already defined cutoff values may not be applicable in all groups of patients, with different prevalence of fibrosis or diagnostic purposes [16].

## 5. VCTE Performance for Cirrhosis Evaluation in HCV Patients

### 5.1. Diagnosis of Cirrhosis by VCTE

One of the greatest benefits of VCTE is the noninvasive diagnosis of cirrhosis. As previously implied, VCTE performs better at evaluating cirrhosis rather than evaluating fibrosis stages [48]. In the Talwalkar meta-analysis [49], the pooled estimates for sensitivity (Se) and specificity (Sp) for cirrhosis were 87% and 91%, respectively. However, the diagnostic threshold bias was an important cause of heterogeneity for pooled results. In 2007, Shaheen et al. [50] provided summary estimates for cirrhosis diagnosis with a Se and Sp of 85.6% and 93.2%, respectively, for LS exceeding 12.5 kPa and AUROC values of 0.95. In another meta-analysis by Stebbing et al. [51], the cutoff value of 15.08 kPa had 84.45% Se and 94.69% Sp. Tsochatzis et al. [52] evaluated the VCTE accuracy for cirrhosis prediction and reported a summary Se and Sp of 83% and 89%, respectively, at a diagnostic threshold of 15 ± 4 kPa. The latest meta-analysis by Ying et al. [53], demonstrated high Se (84%) and Sp (90%) of VCTE for assessing liver cirrhosis in HCV patients. These results suggest that VCTE performs better at ruling out rather than ruling in cirrhosis, with a negative predictive value greater than 90% [35,36,54].

In contrast, regarding US there are conflicting results. The US scoring system (USSS) proposed by Moon et al. [55] seemed to surpass VCTE for the diagnosis of overt cirrhosis, providing 89.2% Se and 69.4% Sp for USSS ≥ 6, while LSM ≥ 17.4 kPa had 77.6% Se and 61.4% Sp. Nevertheless, the Moon study had several limitations, considering that diverse etiologies included in the study provided lower AUROC values for LSM (0.729) than usual. Berzigotti et al. [56] found that among subjects with presumed cirrhosis, US is the better choice to diagnose cirrhosis, whereas VCTE is the preferred method to rule it out. Their combination increased the diagnostic accuracy, contrasting the results of the Zhang [13] and Wang [31] studies.

### 5.2. Screening for Portal Hypertension

Portal hypertension (PH) is a common clinical syndrome of CLD, hemodynamically defined by increased portal venous pressure and a hyperdynamic state [57,58]. In the early, compensated phases of cirrhosis, PH is mainly a result of intrahepatic resistance to portal blood flow due to morphological changes characterized by fibrosis [59]. Subsequently, as the disease progresses, the increase in portal pressure gradient leads to severe complications, consisting of portosystemic collaterals and varices [58]. In cirrhosis, hepatic venous pressure gradient (HVPG) is the standard PH assessment method, but it is invasive and expensive. A HVPG value greater than 10 mmHg represents the threshold for clinically significant portal hypertension (CSPH), a stage where PH complications might arise [58]. For these patients, compensated advanced CLD (cACLD) is an alternative term recommended by the Baveno VI criteria [60], mainly to indicate that the fibrosis progression is a continuum spectrum among asymptomatic patients.

Abdominal US is the primary imaging technique widely used for liver, spleen and portal venous system evaluation, since it can identify PH features, including splenomegaly, portal vein system dilatation, ascites, and portosystemic abdominal collaterals [61,62]. In particular, the incorporation of color and power Doppler enabled the appraisal of the left gastric vein (LGV) hemodynamics, the damping index, and the splenic Doppler pulsatility index [63,64,65]. Of note is the Lee study which reported higher diagnostic accuracy (AUC = 0.873) for splenic arterial resistive index compared to the accuracy of LSM (AUC = 0.745) in a cohort of 47 patients [66]. Nonetheless, existing data is insufficient to recommend Doppler measurements as a trustworthy substitute for HVPG [67].

VCTE proved to be an excellent diagnostic tool for identifying CSPH with a hierarchical summary receiver operating characteristic (HSROC) value of 0.93, reported in the Shi meta-analysis [59]. Table 2 summarizes the results of studies regarding the accuracy of LSM for the prediction of preclinical PH, CSPH, and severe PH (SPH). Carrion et al. [45] were the first to report the significant correlation between LSM and HVPG (Pearson coefficient, 0.84; *p* < 0.001) among patients with HCV recurrence after liver transplant. Over time, these results were confirmed by prospective and retrospective studies in patients with CLD [39,47,59,68,69,70,71,72,73,74]. Even though Schwabl et al. [39] concluded that the etiology was not a significant confounder for the correlation between LSM and HVPG, we decided to emphasize within our table the HCV positive subgroup for integrative purposes [75,76,77,78]. Overall, the AUROC ranged from 0.786 to 0.93 for a threshold of 8 to 8.74 kPa for preclinical PH, AUROC 0.74 to 0.99 for CSPH with the corresponding cutoff values ranging from 13.6 to 21.6 kPa, whilst SPH-related AUROC ranged from 0.721 to 0.92 with the associated cutoff values of 17.6 to 24.5 kPa. These results suggest that, even if the correlation between the two parameters does not allow accurate HVPG estimation, LS has great discriminative power for the presence of CSPH [27]. Recently, a multicenter study of 5648 patients proposed a novel set of cutoff values of <7 and >12 kPa for excluding and diagnosing compensated advanced liver disease. Lowering the dual threshold initially proposed by the Baveno VI consensus provided excellent Se (91%) for ruling out and Sp (92%) for ruling in cACLD, safely reducing the use of LB [60].

### 5.3. Prognostic Significance of Liver Stiffness in Patients with HCV Cirrhosis

There is growing evidence to support the use of VCTE for risk stratification and prognosis [27] even in HCV cirrhosis. In a study of 1457 CHC patients, LS had stronger prognostic value for overall 5-year mortality compared to histological fibrosis staging [79]. In addition, LSM by VCTE has been validated as a prognostic quantitative marker for developing liver related complications, including esophageal varices (EV), variceal bleeding, hepatic decompensation, and HCC [79,80,81,82]. Recent data suggests that liver and spleen stiffness correlate considerably with HVPG among cirrhotic patients. In fact, spleen stiffness seems to be superior to LS for the prediction of PH and can even predict the late recurrence of HCC [83,84,85].

#### 5.3.1. Prediction of Esophageal Varices (EV) and Variceal Hemorrhage by VCTE

In the past years, several studies sought to discover LS accuracy for predicting the presence and size of EV [35,70,75,76,78,86]. In general terms, the greater the LS value—the higher the risk of the patient to present EV and an increased degree of EV, respectively [80]. However, as illustrated by Kim et al. [80], the cutoff values vary widely among studies and VCTE accuracy is still inappropriate to replace HVPG or upper GI endoscopy in screening for EV presence or determining their grade [27,48]. However, it should be mentioned that there were no noninvasive methods that proved to be satisfactorily enough. Even if several studies [64] found that LGV hepatofugal flow substantially correlates with EV, Doppler parameters are still unsuited to be a surrogate for esophagogastroduodenoscopy or HVPG, mostly as a result of significant inter-observer variability [67]. The current reference standard for the detection and classification of EV remains the esophagogastroduodenoscopy procedure, in spite of being an invasive and expensive method [67]. Nonetheless, VCTE should be used as an initial noninvasive method for selecting patients in whom these invasive procedures are indicated [48]. Recent data suggests that the combination between LS, spleen dimensions, and platelet count significantly improves the diagnostic accuracy of EV [87]. In fact, according to the latest recommendations of the Baveno VI guidelines, upper GI endoscopy can be safely avoided among patients with a LS value of <20 kPa and a platelet count greater than 150 G/L [88].

#### 5.3.2. The Prognostic Value of VCTE for HCC Development Prediction

In patients with CLD, abdominal US is the first-line investigation for the detection and characterization of FLLs and the main screening tool for HCC with 51–87% Se and 80–100% Sp [89,90,91]. The add-on of US contrast agents improved the overall diagnostic accuracy of conventional US, offering comparable performance to magnetic resonance imaging or computed tomography for FLLs evaluation [92]. However, even though US significantly improves HCC surveillance, it lacks prognostic power. Increasing evidence implies that noninvasive methods, such as VCTE, are not solely a substitute for LB, but also predictive for liver-related complications, in particular HCC development [48]. It is well known that the degree of fibrosis is by far the strongest risk factor for developing HCC in HCV patients [93]. A decade ago, Masuzaki et al. [94] were the first to describe the relationship between LS and HCC incidence in a Japanese cohort of 866 CHC patients. The hazard ratio (HR) for HCC incidence was 16.7, 20.9, 25.6 and 45.5 for LS values of 10.1–15.0 kPa, 15.2–20.0 kPa, 20.1–25.0 kPa, and >25.0 kPa, respectively (*p* < 0.001). Other longitudinal prospective studies evaluated the prognostic performance of VCTE for the prediction of HCC development in HCV patients, with cutoff values ranging between 12–50 kPa [81,95,96,97,98,99]. In addition, Feier et al. [96] surprisingly found that an IQR exceeding 39% of median LSM is another adequate indicator and essential predictor for the presence of HCC. Nonetheless, in order to confirm whether LS can actually foresee liver-related complications, these results require further validation through prospective studies conducted on large cohorts. In case these results are validated and standardized, VCTE might become an efficient method for the noninvasive screening of patients with CLD, with a possibility to classify them in different risk categories [16]. An interesting point to make is that the elastography parameter already provided effective risk prediction models, especially in patients with chronic hepatitis B infection [100,101,102,103,104]. However, existing literature does not provide any prediction model for HCV-related HCC risk.

Following the availability and efficacy of direct-acting antivirals (DAAs), several studies sought to elucidate their capability of reducing the HCC risk, and whether VCTE might become helpful in objectifying it. Some studies and one meta-analysis reported that the risk of de novo HCC development is similar or even diminished in the subgroup receiving antiviral treatment, compared to the general population [105,106,107,108,109]. However, the absolute risk in patients with cirrhosis remains high, regardless of therapy, which is why this subset of patients should be considered for ongoing HCC surveillance [110]. Elastography facilitates dynamic prediction of HCC, especially before and after the antiviral treatment. In terms of independent risk factors, increased baseline LS and other noninvasive markers of fibrosis, as well as a less than 30% decrease in LS, correlate significantly with the risk of developing HCC [111,112]. In addition, Ioannou et al. [113] developed and internally validated models that estimate the risk of HCC development after DAA therapy, improving HCC surveillance efforts. Nonetheless, their prediction models based on cirrhosis and sustained viral response (SVR) require further international endorsement. In a combined case report–literature review, Strazzulla et al. [114] described a particular case of recurrent HCC after successful DAA treatment in a HCV positive 53-year old patient that received liver transplantation. Although the literature is rather scarce, VCTE may also prove useful in evaluating liver disease progression towards HCC in HCV patients receiving liver transplantation [115].

## 6. VCTE Use for Longitudinal Monitoring in Detecting Fibrosis Regression and Predicting Complication Risk after Achieving Sustained Viral Response

As previously mentioned, the main endpoints in CHC patients are the detection of significant fibrosis (≥F2) and cirrhosis (F4), which have been the definitive indication of antiviral therapy for a long time [27]. However, due to the large availability of highly efficient DAAs, it is expected that significant fibrosis will no longer be a critical decision-making endpoint among these subjects [48].

VCTE, serving as a novel noninvasive method for fibrosis assessment, facilitates the longitudinal evaluation of HCV patients, before and after antiviral treatment. However, fibrosis and PH regression in patients with treated HCV-related cirrhosis is still a debatable subject [116]. Several studies explored the dynamics of LS in patients receiving antiviral therapy (interferon based/interferon-free therapies), concluding that the LS values decreased significantly in those with SVR [111,117,118,119,120,121,122,123,124,125,126,127,128,129]. Most of these studies showed better improvement of LS among patients with higher pre-treatment fibrosis stages [111,117,118,119,120,121,122,129]. However, Persico et al. [124] found that EV of any size anticipated a lack of LS improvement. A study by Chan et al. [125] reported that a baseline elevated ALT was independently associated with a reduction of LS beyond 30%. As assumed by some researchers, this might come as a result of substantial decrease of liver inflammation, rather than fibrosis regression, at the end of the antiviral therapy [116,128,130,131,132]. Nonetheless, several reports showed that liver fibrosis reverses in approximately one third to nearly half of CHC patients [133]. Of note is the D’Ambrosio study, which found significant cirrhosis regression by LB in 61% of individuals with HCV-related cirrhosis [134].

## 7. Controlled Attenuation Parameter (CAP) for the Noninvasive Evaluation of Steatosis in HCV-Infected Patients

Besides fibrosis, steatosis is another common histological feature in HCV patients, especially those infected with genotype 3 [135]. Viral contamination is an independent risk factor for fat accumulation in HCV patients, along with obesity, type II diabetes mellitus, and alcohol consumption. Steatosis was found to be 1.5–2.5 times more prevalent among these subjects than in the general population [136]. In fact, several studies reported that steatosis might increase fibrosis progression and the risk of HCC development while lowering the response rate to antiviral treatment [137,138,139,140]. Therefore, steatosis assessment in HCV positive individuals is of great importance.

At present, abdominal conventional US is the most readily available, simple and cost-effective technique for steatosis appraisal in clinical setting [141]. A 2011 meta-analysis by Hernaez et al. [142] confirmed that B-mode US is a reliable method for steatosis assessment in comparison to liver biopsy. Among 4720 patients, liver US provided 84.8% Se (95% CI: 79.5–88.9), 93.6% Sp (95% CI: 87.2–97.0) and AUROC of 0.93 (95% CI: 0.91–0.95) for moderate to severe steatosis detection. However, its sensitivity lowers when less than 30% of the hepatocytes are affected. Besides, it remains a subjective method, resulting in high variability and low reproducibility [141]. The introduction of the hepatorenal Index (HRI) sought to overcome this drawback, providing excellent diagnostic precision for the diagnosis of steatosis (>5%) with AUROC of 0.99, 100% Se and 91% Sp [143]. Novel quantitative US parameters from radiofrequency data analysis show promising results, surpassing the HRI [144].

Furthermore, numerous studies investigated the use of the novel CAP for steatosis evaluation, as a substitute for the invasive LB [145,146,147]. Several meta-analyses offered consistent results, with AUROC values ranging from 0.81–0.96 for the detection of mild steatosis (≥S1), 0.82–0.90 for moderate steatosis (≥S2), and 0.70–0.97 for severe steatosis (≥S3) [148,149,150]. In 2017, an individual patients’ data meta-analysis, involving 2735 CLD subjects, provided cutoff values of 148 dB/m, 286 dB/m and 280 dB/m for the presence of mild, moderate, and severe steatosis, respectively, using the M probe [23]. However, novel data suggests that optimal cutoff values vary significantly by both probes across different etiologies. Regarding HCV patients, the latest comprehensive meta-analysis could not analyze in great detail this pathology, due to the small cohort and low prevalence of high-grade steatosis [25]. Therefore, additional data concerning this etiology is still needed. Regarding performance, the Moret study found that the hepatorenal B-mode ratio and CAP have comparable power for the diagnosis of steatosis (≥S1), but both lack the ability to discern between moderate to severe steatosis [151].

Moreover, studies show conflicting results with the use of CAP for steatosis evaluation in the context of the new DAA therapy. On one hand, Rout et al. [152] and Ogasawara et al. [153] reported that the CAP score tends to increase in patients treated with DAAs, but these studies could not find an explanation for this phenomenon. On the other hand, two other papers found that DAAs significantly lower hepatic steatosis in chronic HCV patients with fatty liver, while the Sung study noted significant steatosis reduction only in patients with moderate fatty infiltration (S0-S1) at baseline evaluation [154,155,156]. Nevertheless, CAP remains a powerful add-on in the management of HCV patients.

## 8. Advantages and Limitations of VCTE

Although VCTE is increasingly used in daily practice as a noninvasive and efficient method of assessing liver stiffness, it has several limitations. Technically, VCTE cannot be performed in patients with ascites because the elastic waves are not able to penetrate the fluids. Moreover, VCTE is limited by the narrow intercostal space and some obese patients present a challenge in the VCTE examination. In obese patients, the XL probe is required in order to reduce the failure rate [10,26,48,157]. Furthermore, in a multivariate analysis by Castera et al. [20], the only factor associated with failure was obesity (body mass index > 28 kg/m^2^, *p* < 0.001) and VCTE was not successful in 20% of cases. Other factors, such as abdominal wall edema or congestion, can alter the measurements and increase the stiffness, independently of fibrosis.

From another point of view, the main limitations are the need for a dedicated device, which is not always available, and the fact that it is not possible to choose a region of interest for the measurements. Individual factors related to the patient’s condition, such as acute hepatitis, increased transaminases, extrahepatic cholestasis, congestion, and food or excessive alcohol intake could increase liver stiffness, resulting in false positive results [27].

## 9. Concluding Remarks

In the current paper, we have critically reviewed VCTE performance in the assessment of HCV patients, highlighting the advantages of this ultrasound elastographic technique in comparison to conventional US. Besides staging liver fibrosis, the high specificity and negative predictive value of VCTE suggest that it performs better at ruling out cirrhosis rather than diagnosing it. Furthermore, the high hierarchical summary receiver operating characteristic of VCTE in diagnosing CSPH proved the efficacy of this ultrasound elastography method in identifying CSPH. The current range of LS cutoff values for predicting the presence and size of the esophageal varices are wide and standardized values are not available. However, a general rule is that ‘the greater the stiffness, the higher the possibility of esophageal varices and their diameter’. Whilst existing literature suggests that VCTE can be used for HCC risk prediction in other hepatopathies, there are currently no indications for risk prediction in HCV. This would be an important application, as VCTE already allows patient stratification through risk assessment in some instances. One of its upsides that opened a new era in HCV management is that it can be repeated every time it is deemed necessary—before antiviral therapy, in monitoring fibrosis regression after HCV eradication. As such, the advantages of VCTE significantly outweigh those of other surveillance methods.

Our opinion is that HCV patients can greatly benefit from VCTE due to its numerous qualities—rapid, noninvasive, repeatable for longitudinal evaluation and the cost-effectiveness. We propose that further studies should focus on establishing standardized cutoff values of LS for predicting the presence and size of esophageal varices, as well as investigating the potential for predicting HCC risk in HCV patients, which is considered to be of great importance in current clinical practice.

## Figures and Tables

**Table 1:** Performance of LS cutoff values by VCTE for predicting moderate fibrosis (≥F1), significant fibrosis (≥F2), advanced fibrosis (≥F3), and cirrhosis (F4) in chronic HCV infected patients.

Fibrosis Stage	≥F1			≥F2			≥F3			F4		
Study	Cutoff (kPa)	AUROC	Se/Sp (%)	Cutoff (kPa)	AUROC	Se/Sp (%)	Cutoff (kPa)	AUROC	Se/Sp (%)	Cutoff (kPa)	AUROC	Se/Sp (%)
Castera et al. [35] (*n* = 183)	N/S	N/S	N/S	7.1	0.83	67/89	9.5	0.90	73/91	12.5	0.95	87/91
Carrion et al. [45] (*n* = 169)	N/S	N/S	N/S	8.50	0.90	90/81	N/S	0.93	N/S	12.50	0.98	100/87
Ziol et al. [34] (*n* = 327) ^1^	N/S	N/S	N/S	8.80	0.79 0.81	56/91	9.60	0.91 0.95	86/85	14.60	0.97 0.99	86/96
De Ledinghen et al. [36] (*n* = 77) ^2^	N/S	N/S	N/S	4.5	0.72	93.2/17.9	N/S	N/S	N/S	11.8	0.97	100/92.7
Arena et al. [37] (*n* = 161)	N/S	N/S	N/S	7.8	0.91	83/82	10.8	0.99	91/94	14.8	0.98	94/92
Sporea et al. [46] (*n* = 191)	N/S	N/S	N/S	6.8	0.733	59.6/93.3	N/S	N/S	N/S	N/S	N/S	N/S
Nitta et al. [43] (*n* = 165)	N/S	N/S	N/S	7.1	0.87	80.8/80.3	9.6	0.91	87.7/82.4	11.6–16.9	0.93	62.5–91.7/78.9–91.5
Sanchez-Conde et al. [42] (*n* = 100)	N/S	N/S	N/S	7	0.80	76.7/75.4	11	0.93	80/90.6	14	0.99	100/93.5
Reiberger et al. [47] (*n* = 290)	N/S	N/S	N/S	7.2	0.690	73.3/77.4	9.6	0.737	86.9/82.9	12.1	0.904	84.8/86.8
Zarski et al. [38] (*n* = 382)	N/S	N/S	N/S	5.2	0.82	96.6/34.8	N/S	N/S	N/S	12.9	0.93	76.8/89.6
Lupsor et al. [10] (*n* = 1202)	5.3	0.879	84.99/73.21	7.4	0.889	80.32/83/97	9.1	0.941	88.8/88.3	13.2	0.970	93.75/93.31
Schwabl et al. [39] (*n* = 188)	N/S	N/S	N/S	7.2	0.852	N/S	N/S	N/S	N/S	14.5	0.852	N/S
Yoneda et al. [40] (*n* = 102)	5.5	0.838	84.6/71.4	7.8	0.906	77.9/90.0	10.4	0.952	88.1/91.1	11.3	0.907	90.0/83.8
Njei et al. [41] * (*n* = 756) ^2^	N/S	N/S	N/S	4.5–7.2	N/S	97/64	N/S	N/S	N/S	11.8–14.6	N/S	90/87

* Meta-analysis, N/S = not specified. ^1^ The Ziol study investigated the effect of biopsy length on the diagnostic performance of LSM, providing AUROC values for small and large biopsies, respectively. ^2^ These studies evaluated the use of VCTE in HIV-HCV coinfection.

**Table 2:** Accuracy of LSM for the prediction of preclinical PH, CSPH, and SPH.

Grade of Portal Hypertension (PH)		Preclinical PH (≥5 mmHg)			Clinically Significant PH (≥10 mmHg)			Severe PH (≥12 mmHg)		
**Study**	Correlation Coefficient	Cutoff (kPa)	AUROC	Se/Sp (%)	Cutoff (kPa)	AUROC	Se/Sp (%)	Cutoff (kPa)	AUROC	Se/Sp (%)
Carrion et al. [45] (*n* = 129) ^1^*	0.84	8.74	0.93	90/81	N/S	0.94	N/S	N/S	N/S	N/S
Vizzutti et al. [75] (*n* = 61) *	0.81	N/S	N/S	N/S	13.6	0.99	97/92	17.6	0.92	94/81
Bureau et al. [76] (*n* = 150) *	0.858	N/S	N/S	N/S	21	0.971	89.9/93.2	N/S	N/S	N/S
Lemoine et al. [77] (*n* = 44) *	0.46	N/S	N/S	N/S	20.5	0.76 ± 0.07	63/70	N/S	N/S	N/S
Sanchez-C. et al. [78] (*n* = 38) ^2^*	0.46	N/S	N/S	N/S	14	0.80	92.86/50	23	0.80	82.61/66.67
Reiberger et al. [47] (*n* = 390)	0.838	8	0.830	95.3/71	18	0.892	80.3/86.9	20	0.899	84.4/86.5
Llop et al. [68] (*n* = 52) ^#^	0.646	N/S	N/S	N/S	Rule out: 13.6 Rule in: 21	0.857	88/61 42/100	N/S	N/S	N/S
Schwabl et al. [39] (*n* = 188) ^#^	0.846	N/S	N/S	N/S	16.1	0.957	94.8/86.9	N/S	N/S	N/S
Hong et al. [69] (*n* = 59) ^#^	0.496	N/S	N/S	N/S	21.95	0.851	82.5/73.7	24.25	0.877	82.9/70.8
Augustin et al. [70] (*n* = 40) ^#^	N/S	N/S	N/S	N/S	25	N/S	65/93	N/S	N/S	N/S
Kitson et al. [71] (*n* = 95) ^#^	N/S	N/S	N/S	N/S	29.0	0.900	71.9/100	N/S	N/S	N/S
Zykus et al. [72] (*n* = 107) ^#^	0.75	N/S	N/S	N/S	17.4	0.949	88/87.5	20.6	0.915	82.8/80
Procopet et al. [73] (*n* = 55) ^#^	0.699	N/S	N/S	N/S	19.0 ± 13.3	0.926	N/S	N/S	N/S	N/S
Kumar et al. [74] (*n* = 326)	0.361	N/S	0.786	N/S	21.6	0.74	79/67	N/S	0.721	N/S

* Data displayed only for HCV infected patients. ^#^ mixed etiologies, mostly viral or alcoholic liver disease. ^1^ The Carrion study used the threshold ≥6 mmHg for the diagnosis of PH. ^2^ The Sanchez-Conde study evaluated correlation between LSM and HVPG in HIV-HCV coinfection.
